# Osthole Improves Spatial Memory Deficits in Rats via Hippocampal **α**_1_-Adrenergic and D_**1**_/D_**2**_ Receptors

**DOI:** 10.1155/2013/273682

**Published:** 2013-02-26

**Authors:** Li-Wei Lin, Yueh-Hsiung Kuo, You Cheng Hseu, Chia-Wen Tsai, Ming-Tsuen Hsieh, Shiu Ching Chen, Chi-Rei Wu

**Affiliations:** ^1^The School of Chinese Medicines for Post-Baccalaureate, I-Shou University, No.8, Yida Road, Yanchao Township, Kaohsiung County 82445, Taiwan; ^2^The Department of Chinese Pharmaceutical Sciences and Chinese Medicine Resources, College of Pharmacy, China Medical University, No.91, Hsueh Shih Road, Taichung 40402, Taiwan; ^3^Department of Cosmeceutics, College of Pharmacy, China Medical University, No.91, Hsueh Shih Road, Taichung 40402, Taiwan; ^4^Department of Nutrition, China Medical University, No.91, Hsueh Shih Road, Taichung 40402, Taiwan; ^5^Department of Health, Taichung Hospital, The Executive Yuan, No.199, San Min Road, Taichung 40403, Taiwan

## Abstract

The present study evaluated the effect of osthole, an active ingredient isolated from *Cnidium monnieri* L. Cusson, on spatial memory deficits caused by central neurotoxins using the Morris water maze in rats. The involvement of catecholaminergic receptors on the memory-enhancing effect of osthole in rat hippocampus was further investigated by intrahippocampal injection of catecholaminergic receptor antagonists. Intracisternal injection of osthole (10 **μ**g/brain) improved the spatial performance and working memory impairments caused by the catecholaminergic neurotoxin 6-hydroxydopamine. No significant differences in swimming speeds were observed among sham, neurotoxin-induced, and osthole-treated groups. Intracisternal osthole injection also attenuated the spatial performance and working memory impairments caused by the *α*_1_ receptor antagonist phenoxybenzamine, the D_1_ receptor antagonist SCH 23390, and the D_2_ receptor antagonist sulpiride. Therefore, we demonstrated that the effect of osthole on improving spatial memory deficits may be related to the activation of hippocampal *α*_1_ and D_1_/D_2_ receptors.

## 1. Introduction

Learning acquisition usually involves the activation of neurotransmitters such as acetylcholine, noradrenaline, dopamine, and serotonin [1]. According to a meta-analysis by Myhrer on four behavioral tasks [2], acetylcholinergic and dopaminergic activities have a high influence on learning and memory. It is well known that the central acetylcholinergic neuronal system plays an important role in learning and memory in humans and animals [3]. The central cholinergic neurotoxin, ethylcholine mustard aziridinium ion (AF64A), causes the loss of central cholinergic neurons and the impairment of cognitive performance measured with the water maze and inhibitory avoidance task [4]. The central catecholaminergic system also plays an important role in learning and memory. Intracisternal 6-hydroxydopamine (6-OHDA) produces long-term presynaptic catecholaminergic deficits and cognitive function impairments [5, 6]. Moreover, the serotonergic system has a moderate influence on learning and memory [2]. Intracisternal 5,7-dihydroxytryptamine (DHT) also produces long-term presynaptic serotonergic deficits and cognitive function impairments [7]. The serotonergic synthesis inhibitor para-chlorophenylalanine (PCPA) also impairs memory processes in various tasks [8].

Osthole (7-methoxy-8-isopentenoxycoumarin) is a major active coumarin ingredient of *Cnidium monnieri* L. Cusson (*Umbelliferae*, abbreviated as CM) that is used in traditional Chinese medicine to treat Kidney Yang deficiency, consisting of fatigue, senescence, and impotence symptoms [9]. Our previous study found that CM and osthole alleviated scopolamine-induced amnesia in male and female rats [10, 11]. It is possible that the antiamnesic effects of osthole are partially due to the activation of the adrenal gland and central nervous system, but these effects are not due to the activation of peripheral nervous system [10, 11]. In response to other reports indicating that osthole protects against hippocampal damage induced by middle cerebral artery occlusion and facilitates glutamate release from hippocampal nerve terminal in rats [12, 13], the present study explored the memory-enhancing effects of osthole. We first evaluated the effects of osthole on spatial memory deficits caused by intracisternal administration of the cholinergic neurotoxin AF64A, the catecholaminergic neurotoxin 6-OHDA, and the serotonergic neurotoxin DHT in rats. To further elucidate osthole's mechanism of action on memory function, we investigated whether osthole attenuates the spatial memory deficits caused by intrahippocampal administration of noradrenergic or D_1_/D_2_ receptor antagonists.

## 2. Materials and Methods

### 2.1. Chemicals

Osthole, supplied by Yueh-Hsiung Kuo, was isolated from CM and identified with physical and spectral methods [14]. Osthole was dissolved with 0.01% ethanol using a dosage method described previously [10]. 6-OHDA, acetylcholine mustard hydrochloride, DHT, phenoxybenzamine hydrochloride (PHEN), (±)-propranolol hydrochloride (PROP), (+)-SCH 23390 hydrochloride (SCH), (±)-sulpiride (SUL), and yohimbine hydrochloride (YOH) were purchased from Sigma-Aldrich (St. Louis, MO, USA). 6-OHDA and DHT were dissolved in physiological saline containing 0.5% (w/v) ascorbic acid [5, 6]. AF64A was freshly prepared by dissolving acetylcholine mustard hydrochloride in physiological saline according to our previous technique [15]. The pH was adjusted to 7.4 with NaHCO_3_ and the solution was maintained at room temperature for 1 h [15]. PHEN, PROP, SCH, SUL, and YOH were dissolved in physiological saline. Osthole was intracisternally administered in a volume of 20 *μ*L/brain.

### 2.2. Animals

Male Sprague-Dawley rats, weighing 200–250 g, were housed in groups of six with free access to food (supplied and manufactured by Fwusow Industry Co. Ltd., Taiwan) and water in a regulated environment (23 ± 1°C), with a 12 h light-dark cycle (light period: 08 : 00 to 20 : 00 h) for at least 1 week before the start of the experiment. The rats were randomly assigned into groups of 8 to 10 animals. The drug administration and behavioral assays were performed using a double-blind method. This protocol was approved by the Committee on Care and Use of Laboratory Animals of China Medical University.

### 2.3. Surgery

Approximately 14 days prior to the initiation of the behavioral experiments, rats were anesthetized with sodium pentobarbital (45 mg/kg intraperitoneally) and mounted in a stereotaxic frame (Stoelting, IL, USA) with lambda and bregma in the same horizontal plane. Rats were implanted with cannulas (12 mm, 23 gauge) aimed at sites above the lateral ventricle or hippocampus. For intracisternal injection, a hole was drilled in the skull at coordinates AP −0.8 mm, ML −1.5 mm. The cannula was inserted to a vertical depth of 3.6 mm below the dura mater [16]. For intrahippocampal injection, two holes were drilled in the skull at coordinates AP −5.0 mm, ML ± 3.6 mm. The cannula was inserted to a vertical depth of 4.0 mm below the dura mater [16]. These cannulas and two anchoring screws were fixed in positions with dental cement. Postoperative care included a single subcutaneous injection of 5 mL sterile saline, a single intramuscular injection of 0.2 mL penicillin, and a heating pad under the cage for the first 2 h after surgery.

### 2.4. Microinjection Procedure

Before intracisternal injection, the animal was restrained by hand, and the cannula stylet was removed and replaced with a 30-gauge injection needle, which was connected to the 25 *μ*L Hamilton syringe (Model 702RN, Reno, NV, USA) by a short piece of polyethylene tubing. For intrahippocampal injection, a 30-gauge injection needle was connected to the 10 *μ*L Hamilton syringe (Model 701RN, Reno, NV, USA) by a short piece of polyethylene tubing. The needle was inserted 0.5 mm beyond the tip of the cannula; 20 *μ*L of vehicle or osthole was injected into the lateral ventricle, or 5 *μ*L of induced drug was injected into the hippocampus, with an infusion pump (KDS310 syringe pump, KD Scientific Inc., Holliston, MA, USA) at a rate of 1 *μ*L/min. Before removal, the needle was left in place for another 2 min to allow diffusion of osthole or induced drugs into the surrounding tissue.

### 2.5. Behavioral Measurement

To assess spatial learning and memory function, the rats were tested in a Morris water maze (MWM). The MWM was a black circular stainless steel pool (with a diameter of 165 cm and a height of 60 cm) filled with 23 ± 1°C water to a depth of 35 cm. The maze was divided geographically into four equal quadrants and included release points in each quadrant. The position of the white rat in the black pool was recorded by a video camera and an automated video tracking system equipped with EthoVision XT software (Noldus Information Technology, Leesburg, VA, USA). The swim path, escape latency, and swimming speed were recorded for each trial.

Each rat performed four trials per day for 4 consecutive days to find the hidden platform. A hidden platform made of Plexiglas (with a diameter of 10 cm), submerged 1.0 cm below the water surface, was situated in the center of the northeast quadrant and remained stable during the four days of spatial learning. Each trial began by placing a rat into one of the four quadrants of the pool, facing the wall of the tank. The daily order of the entries into individual quadrants was randomized so that all four quadrants were used once in a series of four trials every day. Each trial was terminated as soon as the rat climbed onto the hidden platform or when 120 s had elapsed. A rat was allowed to stay on the platform for 30 s. Rats that did not find the platform within 120 s were put on the platform by the experimenter and were also allowed to stay there for 30 s. Then, the rat was taken from the platform, and the next trial began after 30 s. After completion of the fourth trial, each rat was kept warm for one hour and returned to its home cage. All tests were conducted between 09 : 00 and 18 : 00. Escape latencies (i.e., the time to reach the platform) were compared between groups.

Twenty-four hours after the last session, the probe test was performed to measure reference memory. For the probe test, the Plexiglas platform was removed from the pool. Each rat was released from the quadrant opposite to where the platform had been located. The probe test measured the time (up to 60 s) and distance spent searching for the platform in the quadrant where the platform had been located during training.

The day after the probe test, working memory was tested using the MWM. In the first trial (acquisition), rats had to find the platform, now located in a new position, and were allowed to remain on the platform for 30 s before they were returned to the home cage. In the second trial (retrieval) 4 h later, rats began the maze from a different quadrant and had to find the platform located in the same position as in the previous trial.

### 2.6. Intracisternal Injection with Neurotoxins

Rats with a cannula were divided into vehicle, neurotoxin-treated, and osthole-plus-neurotoxin-treated groups. Then neurotoxin-treated groups and osthole-plus-neurotoxin-treated groups were divided into AF64A, DHT, and 6-OHDA subgroups. AF64A (3 nmol/brain) was intracisternally administered to rats, and spatial learning in the MWM was performed 10 days after neurotoxin injection [4, 14]. DHT (250 *μ*g/brain) or 6-OHDA (250 *μ*g/brain) was intracisternally administered to rats, and spatial learning in the MWM was performed 14 days after neurotoxin injection [5, 7, 15].

Fifteen minutes before each spatial learning session and the first trial of the working memory test, vehicle and neurotoxin-treated groups received an intracisternal injection of vehicle. The osthole-plus-neurotoxin-treated group received an intracisternal injection of osthole (10 *μ*g/brain) [10]. No infusion was given before either the probe test or the second trial of the working memory test.

### 2.7. Intrahippocampal Injection of Noradrenergic Receptor Antagonists

Rats with three cannulas were divided into vehicle, noradrenergic-antagonist-treated, and osthole-plus-noradrenergic antagonist-treated groups. Then noradrenergic-antagonist-treated groups and osthole-plus-noradrenergic antagonist-treated groups were divided into PHEN, PROP, and YOH subgroups.

Fifteen minutes before each spatial learning session and the first trial of the working memory test, vehicle groups received intracisternal and bilateral intrahippocampal injections of vehicle. Noradrenergic-antagonist-treated groups received an intracisternal injection of vehicle and bilateral intrahippocampal injections of PHEN, PROP, or YOH (80 ng/side) [17–19]. The osthole-plus-noradrenergic-antagonist-treated group received an intracisternal injection of osthole (10 *μ*g/brain) and bilateral intrahippocampal injections of PHEN, PROP, or YOH (80 ng/side). No infusion was given before either the probe trial or the second trial of working memory test.

### 2.8. Intrahippocampal Injection of Dopaminergic Receptor Antagonists

Rats with three cannulas were divided into vehicle, dopaminergic-antagonist-treated, and osthole-plus-dopaminergic-antagonist-treated groups. Then dopaminergic-antagonist-treated groups and osthole-plus-dopaminergic-antagonist-treated groups were divided into SCH and SUL subgroups.

Fifteen minutes before each spatial learning session and the first trial of the working memory test, vehicle groups received intracisternal and bilateral intrahippocampal injections of vehicle. Dopaminergic-antagonist-treated groups received an intracisternal injection of vehicle and bilateral intrahippocampal injections of SCH or SUL (80 ng/side) [20, 21]. The osthole-plus-dopaminergic-antagonist-treated group received an intracisternal injection of osthole (10 *μ*g/brain) and bilateral intrahippocampal injections of SCH or SUL (80 ng/side). No infusion was given before either the probe trial or the second trial of working memory test.

### 2.9. Histology

**Figure 1:**
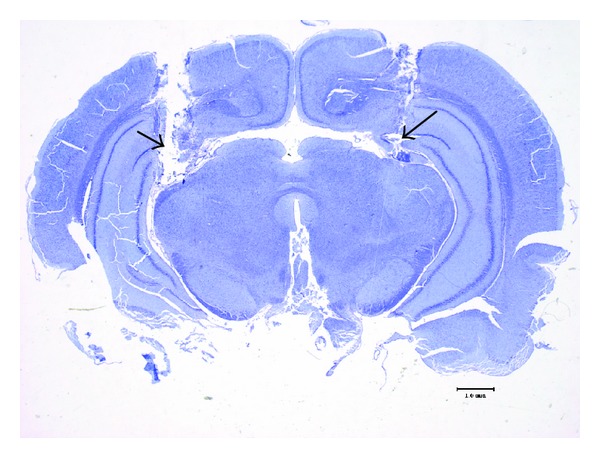
Photomicrograph of hippocampus, showing cannula termination area.

After completion of the behavioral experiments, the rats given intrahippocampal injections were anesthetized with sodium pentobarbital (45 mg/kg) and their brains were perfused with 10% formalin solution through their left cardiac ventricle. After the brains were removed, they were stored in 10% formalin solution and then sectioned into 25-*μ*m slices. All sliced sections were stained with cresyl violet. The placement of the cannula was verified histologically, and the stained sections showed that the cannulas were successfully located in the hippocampus (Figure 1).

### 2.10. Statistical Analysis

For spatial learning in the MWM, the escape latency for each rat was obtained by averaging results from the four trials prior to analysis. The parameters were analyzed using a one-way repeated-measures analysis of variance (ANOVA), with treatment defined as the between-subjects variable and day as the within-subject variable. If the treatment effect was significant, post hoc comparisons were conducted with Dunnett's test (family error rate *P* < 0.05 was considered statistically significant). For the probe test and the working memory test of the MWM, swimming speed and escape latency for each rat were expressed as the mean ± standard errors (SEM) and analyzed with a one-way ANOVA, followed by Dunnett's test. When the probability (*P*) was less than 0.05, the difference was considered to be significant.

## 3. Results and Discussion

**Figure 2:**
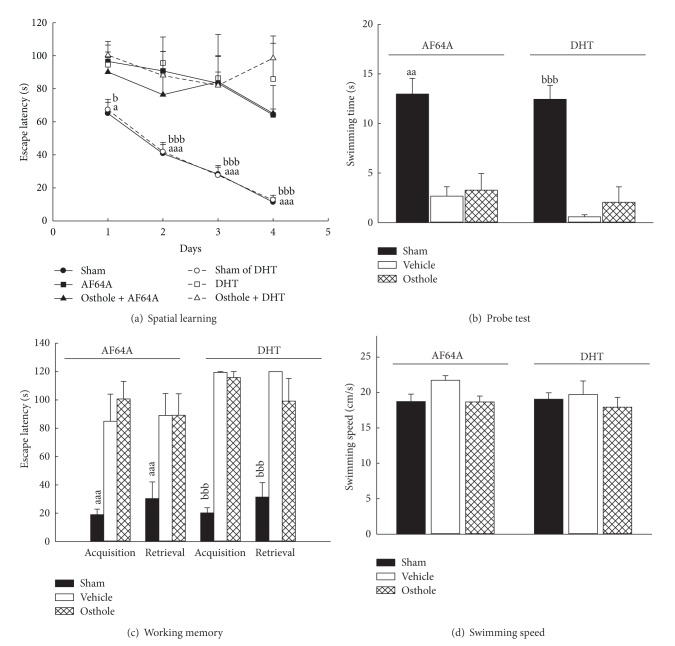
Effects of osthole (icv, 10 *μ*g/brain) on the performance impairment induced by acetylcholine mustard (AF64A, icv, 3 nmol/brain) or 5,7-dihydroxytryptamine (DHT, icv, 250 *μ*g/brain) in rats. (a) The escape latency on the four-day spatial learning, (b) the swimming time on the probe test which spent in the quadrant where the platform was, (c) the escape latency on the two trials of working memory version, and (d) the swimming speed on the probe test in the Morris water maze. ^a^ *P* < 0.05, ^aa^ *P* < 0.01, and ^aaa^ *P* < 0.001, compared with AF64A (vehicle) group. ^b^ *P* < 0.05, ^bbb^ *P* < 0.001, compared with DHT (vehicle) group.

Our previous report indicated that systemic and intracisternal administration of osthole reversed the impairment of behavioral performance in scopolamine-treated rats [10]. Therefore, the first aim of this study was to explore the effect of intracisternally administered osthole on spatial memory deficits in rats treated with the acetylcholinergic neurotoxin AF64A. It is evident that the central cholinergic neuronal system plays an important role in learning and memory processes [3]. AF64A, a neurotoxic derivative of choline, significantly decreases hippocampal acetylcholine contents by producing long-term presynaptic cholinergic deficits, thus resulting in impaired learning performance in rats [4]. These performance deficits have been alleviated by treatment with cholinergic agents such as tacrine, huperzine A, or donepezil [22, 23]. The present study showed that intracisternal injection of AF64A impaired spatial learning during the four-day training in the MWM (*P* < 0.001, Figure 2(a)) and also impaired performance in the probe test and the working memory test of the MWM (*P* < 0.01, *P* < 0.001, Figures 2(b) and 2(c)). Consistent with another study that reported that AF64A, which is a selective cholinergic neurotoxin at low doses (1.5–5 nmol), did not alter spontaneous locomotor activity [4], we found that AF64A (3 nmol) did not alter swimming speed in the pool (*P* > 0.05, Figure 2(d)). However, at higher doses (>7.5 nmol), AF64A is a nonselective neurotoxin and decreases spontaneous activity via the dysfunction of other neural systems such as monoaminergic systems [24, 25]. Thus, AF64A at the dosage used here (3 nmol) 10 days after intracisternal injection, consistent with the other report that from 7 to 21 days after intracisternal injection of AF64A at greater than 3 nmol [4, 22, 23], also caused deficits in spatial learning mainly through the destruction of cholinergic presynaptic markers (such as ChAT and high-affinity choline uptake), resulting in decreased central acetylcholinergic activities. Inconsistent with our previous report on the reversal of scopolamine-induced amnesia [10, 11], intracisternal injection of osthole did not reverse performance deficits caused by the cholinergic toxin AF64A (*P* > 0.05, Figures 2(a)–2(c)). This discrepancy may be explained by the differences in the mechanism of induced drugs (template receptor blockade versus permanent neuronal damage) or by the indirect modulation of central cholinergic activity by other neurotransmitters. A recent report indicated that osthole facilitates glutamate release from hippocampal nerve terminals in rats [12]. Moreover, some compounds improve memory impairment caused by scopolamine via other neurotransmitter systems [26–28]. Based on our previous report and the present results, we suggest the memory-enhancing effects of osthole might be due to the modulation of central acetylcholinergic activity via the activation of other central neuronal systems.

**Figure 3:**
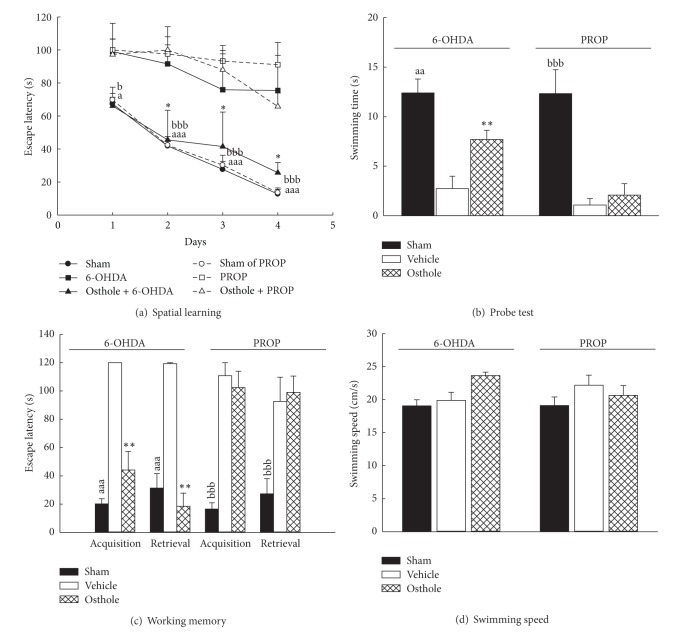
Effects of osthole (icv, 10 *μ*g/brain) on the performance impairment induced by 6-hydroxydopamine (6-OHDA, icv, 250 *μ*g/brain) or propranolol (PROP, 80 ng/side) in rats. (a) The escape latency on the four-day spatial learning, (b) the swimming time on the probe test which spent in the quadrant where the platform was, (c) the escape latency on the two trials of working memory version, and (d) the swimming speed on the probe test in the Morris water maze. ^a^ *P* < 0.05, ^aa^ *P* < 0.01, and ^aaa^ *P* < 0.001, compared with 6-OHDA (vehicle) group. ^b^ *P* < 0.05, ^bbb^ *P* < 0.001, compared with PROP (vehicle) group. *P < 0.05, **P < 0.01, compared with 6-OHDA (vehicle) group.

Many researchers have focused on the interactions between the central cholinergic system and other neurotransmitter systems, such as the serotonergic and catecholaminergic systems in the neural basis of cognitive function [21, 29, 30]. Thus, we further investigated the effects of intracisternal injection of osthole against spatial memory deficits in rats caused by the monoaminergic neurotoxins DHT or 6-OHDA. The serotonergic system is also implicated in learning and memory processes [31]. Previously, intracisternal injection of DHT was used to produce a widespread depletion of brain serotonin and was shown to cause performance deficits in learning and memory tasks until 270 days after intracisternal injection [7, 32]. The present study also showed that intracisternal DHT impaired spatial learning during the four-day training on the MWM, the probe test, and the working memory test of the MWM (*P* < 0.001, Figures 2(a)–2(c)). DHT did not alter swimming speed in the pool (*P* > 0.05, Figure 2(d)), although DHT causes nonspecific behavioral effects, such as motivational or motor effects, via its serotonergic depletion [33]. Our data further illustrated that intracisternal injection of osthole did not reverse performance deficits caused by the serotonergic toxin DHT (*P* > 0.05, Figures 2(a)–2(c)). Alternatively, the dopaminergic system is also implicated in learning and memory processes [34] and the noradrenergic system plays an important modulatory role in memory consolidation of emotionally arousing tasks [35]. Intracisternal injection of 6-OHDA and intraperitoneal injection of DSP-4 have been shown to induce a widespread depletion of brain catecholamines and cause learning deficits in learning and memory tasks [5, 6]. The present study also showed that intracisternal injection of 6-OHDA impaired spatial learning during the four-day training on the MWM, the probe test, and the working memory test of the MWM (*P* < 0.01, *P* < 0.001, Figures 3(a), 3(b), and 3(c)) but did not alter swimming speed in the pool (*P* > 0.05, Figure 3(d)). Our data further illustrated that intracisternal injection of osthole reversed the performance deficits caused by the catecholaminergic toxin 6-OHDA (*P* < 0.05 and *P* < 0.01 for the spatial learning and probe test, *P* < 0.001 for the working memory test, Figures 3(a), 3(b), and 3(c)) but did not affect swimming speed (*P* > 0.05, Figure 3(d)). Therefore, we suggest that intracisternal injection of osthole reverses the spatial memory deficits caused by 6-OHDA, but not AF64A or DHT; therefore, the modulating effects of osthole on memory function might be mainly through the central catecholaminergic system.

**Figure 4:**
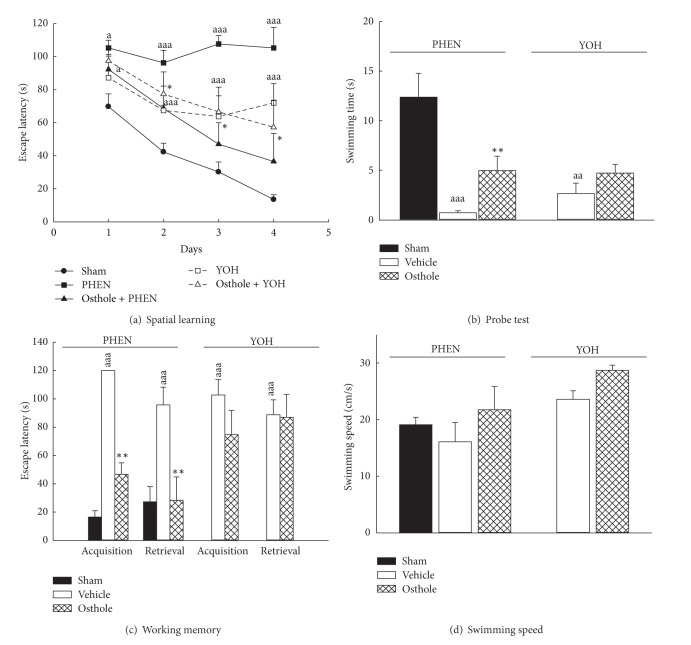
Effects of osthole (icv, 10 *μ*g/brain) on the performance impairment induced by bilaterally intrahippocampal injection of phenoxybenzamine (PHEN, 80 ng/side) or yohimbine (YOH, 80 ng/side) in rats. (a) The escape latency on the four-day spatial learning, (b) the swimming time on the probe test which spent in the quadrant where the platform was, (c) the escape latency on the two trials of working memory version, and (d) the swimming speed on the probe test in the Morris water maze. ^a^ *P* < 0.05, ^aa^ *P* < 0.01, and ^aaa^ *P* < 0.001, compared with sham group. *P < 0.05, **P < 0.01, compared with PHEN (vehicle) group.

According to some reports about the role of central neurotransmitter systems on memory function, the integrity and convergence of sepal noradrenergic, mesocorticolimbic dopaminergic, and septohippocampal acetylcholinergic neuronal inputs to the hippocampus are critical for memory function [19, 36, 37]. Our present histological stain found that the cannula tips were located in the hippocampal CA3 area (Figure 1) and many neurons from the dentate gyrus and the above areas projected into this area. Noradrenergic receptors are mainly classified into the *α* and *β* receptor subtypes. *α*_1_ and *β* receptors participate in processing stress-related information, and the nonselective *β* receptor antagonist PROP, the *α*_1_ receptor antagonist PHEN, and the *α*_2_ receptor antagonist YOH have been shown to impair behavioral performance [17–19]. The present study also showed that intrahippocampal injection of PROP, PHEN, or YOH impaired spatial learning during the four-day training on the MWM, the probe test, and the working memory test of MWM (*P* < 0.01, *P* < 0.001, Figures 3 and 4) but did not alter swimming speed in the pool (*P* > 0.05, Figures 3 and 4). Furthermore, studies have indicated that paeoniflorin improved scopolamine-induced learning impairment through the activation of noradrenergic receptors [27, 28]. Intracisternal injection of osthole significantly attenuated spatial learning deficits shown during the four-day training on the MWM, the probe test, and the working memory test of the MWM caused by intrahippocampal injection of the *α*_1_ receptor antagonist PHEN (*P* < 0.05 for spatial learning, *P* < 0.01 for the probe test and the working memory test, Figures 4(a)–4(c)), but not caused by intrahippocampal injection of PROP (*P* > 0.05, Figures 3(a)–3(c)) or YOH (*P* > 0.05, Figures 4(a)–4(c)).

**Figure 5:**
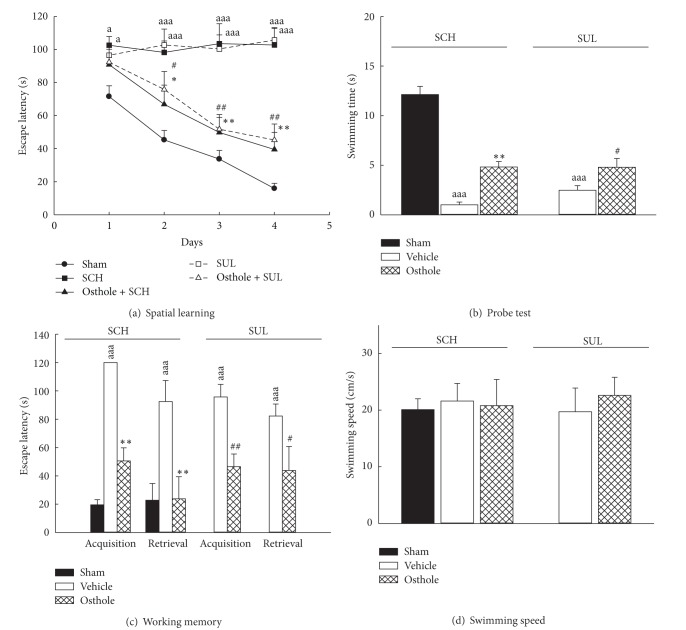
Effects of osthole (icv, 10 *μ*g/brain) on the performance impairment induced by bilaterally intrahippocampal injection of SCH-23390 (SCH, 80 ng/side) or sulpiride (SUL, 80 ng/side) in rats. (a) The escape latency on the four-day spatial learning, (b) the swimming time on the probe test which spent in the quadrant where the platform was, (c) the escape latency on the two trials of working memory version, and (d) the swimming speed on the probe test in the Morris water maze. ^a^ *P* < 0.05, ^aa^ *P* < 0.01, and ^aaa^ *P* < 0.001, compared with sham group. *P < 0.05, **P < 0.01, compared with SCH (vehicle) group. ^#^ *P* < 0.05, ^##^ *P* < 0.01, compared with SUL (vehicle) group.

Dopaminergic receptors are classified into several subtypes. D_1_ and D_2_ receptor subtypes have been reported to play an important role in aversive and rewarded memory formation, and the D_1_ receptor antagonist SCH and the D_2_ receptor antagonist SUL have been shown to impair behavioral performance [20, 21]. We also found that intrahippocampal injection of SCH or SUL impaired spatial learning during the four-day training on the MWM, the probe test, and the working memory test of MWM (*P* < 0.001, Figure 5) but did not alter swimming speed in the pool (*P* > 0.05, Figure 5). Intracisternal injection of osthole significantly attenuated spatial learning deficits caused by intrahippocampal injection of the D_1_ receptor antagonist SCH (*P* < 0.01, Figures 5(a), 5(b), and 5(c)) and the D_2_ receptor antagonist SUL (*P* < 0.05, *P* < 0.01, Figures 5(a)–5(c)). From our previous report [10] and the present data, this modulating effect of osthole on memory function might be related to an increase in central neuronal activity at noncholinergic heteroreceptors such as postsynaptic *α*_1_ and D_1_/D_2_ receptors.

In this study, we found that osthole reversed spatial performance impairments caused by the catecholaminergic neurotoxin 6-OHDA and attenuated spatial performance impairments caused by the *α*_1_ receptor antagonist PHEN, the D_1_ receptor antagonist SCH, and the D_2_ receptor antagonist SUL. Moreover, our previous report indicated that systemic and intracisternal administration of osthole reversed behavioral performance impairments in scopolamine-treated rats [10]. Hence, the beneficial effect of osthole on memory function might be mainly due to the activation of the central catecholaminergic system via postsynaptic *α*_1_ and D_1_/D_2_ receptors. Alternatively, Wang et al. indicated that osthole facilitates glutamate release from hippocampal nerve terminals in rats [12]. Moreover, another report indicated that glutaminergic, GABAergic, and cholinergic septohippocampal neurons contribute to spatial learning and memory stabilization [38]. To better understand the detailed mechanisms underlying osthole-induced improvements in memory functions, additional investigations on the interactions among catecholaminergic, GABAergic, and glutaminergic systems will be necessary.
